# Data Augmentation with Suboptimal Warping for Time-Series Classification

**DOI:** 10.3390/s20010098

**Published:** 2019-12-23

**Authors:** Krzysztof Kamycki, Tomasz Kapuscinski, Mariusz Oszust

**Affiliations:** Department of Computer and Control Engineering, Rzeszow University of Technology, W. Pola 2, 35-959 Rzeszow, Poland; kkamycki@kia.prz.edu.pl (K.K.); tomekkap@kia.prz.edu.pl (T.K.)

**Keywords:** multivariate time-series, data augmentation, time-series classification, machine learning

## Abstract

In this paper, a novel data augmentation method for time-series classification is proposed. In the introduced method, a new time-series is obtained in warped space between suboptimally aligned input examples of different lengths. Specifically, the alignment is carried out constraining the warping path and reducing its flexibility. It is shown that the resultant synthetic time-series can form new class boundaries and enrich the training dataset. In this work, the comparative evaluation of the proposed augmentation method against related techniques on representative multivariate time-series datasets is presented. The performance of methods is examined using the nearest neighbor classifier with the dynamic time warping (NN-DTW), LogDet divergence-based metric learning with triplet constraints (LDMLT), and the recently introduced time-series cluster kernel (NN-TCK). The impact of the augmentation on the classification performance is investigated, taking into account entire datasets and cases with a small number of training examples. The extensive evaluation reveals that the introduced method outperforms related augmentation algorithms in terms of the obtained classification accuracy.

## 1. Introduction

The ubiquity of interconnected sensors that record data over time result in collections of sequentially ordered multidimensional data points. Consequently, the classification of multivariate time-series (MTS) requires taking into account multidimensional features, their relationship with subsequences of features that belong to the same time-series, and possible nonlinear distortions in time scale [1]. The last problem involves sequences of different lengths, which are typical in applications capturing events that occur at different speeds. For example, sign language gestures of the same meaning can be performed differently, depending on the mood or intentions of the signer [2]. This applies to time-series captured by sensors in most action recognition problems [3]. Since the classification of relatively small datasets may lead to overfitting, methods that generate synthetic data examples are desired. Data augmentation techniques are more popular in the computer vision field in which learning images are subjected to different transformation approaches, such as rotation, scaling, or noise injection [4]. Also, datasets which contain sets of multidimensional data points can be augmented using methods creating synthetic minority class examples [5]. They introduce new data samples between pairs of training samples. However, data augmentation of time-series seems more challenging than the augmentation of image data or sets of multidimensional points, as new data points cannot be created in space between time-series. That space is often warped and operators that reflect rotation or scaling of an image may damage the information carried by a sequence of captured events stored in a time-series. Such a sequence has meaning and its distortion may lead to the classification errors [6]. Therefore, time-series augmentation techniques generate new examples by randomly stretching, shrinking, removing their parts [4], or perturbing them [7]. Also, weighted aligned averages [8,9] or generative models [10] are used.

In this paper, a new time-series example is created in the warped space between time-series using a suboptimal alignment. The suboptimality is caused by constraining the warping path. This allows the method to generate synthetic examples that enrich the pool of training data and often create new boundaries between classes. The method is designed for data captured by a variety of sensors [1] and modifies the alignment provided by the Dynamic Time Warping (DTW) [11].

The major contributions of this work are a novel method for the generation of synthetic time-series to augment sparse datasets and a comprehensive evaluation of the approach and related techniques on demanding MTS datasets using representative classifiers.

The rest of this paper is arranged as follows. Section 2 reviews previous work on augmentation of time-series datasets, Section 3 introduces the proposed technique, and Section 4 presents the extensive comparative evaluation of the method with related approaches. Finally, Section 5 concludes the paper and indicates possible directions for future work.

## 2. Related Work

The data augmentation methods aim to provide new data samples that cover unoccupied feature space of the considered class, assuming that this would improve the classification performance. Such simulated data samples are assigned with class labels of the samples used for the augmentation. As data generation techniques rely on linear transformations [5,12], the synthetic time-series generation can be particularly challenging due to frequent nonlinear transformations in time scale or the need for methods that create a new example preserving sufficient information to identify the class.

Simple approaches to time-series generation involve a selection of a time window in a time-series and warping it randomly by removing or adding some data points [4]. Similarly, cropping, or window slicing, in which a part of a time-series is removed can be also used [4]. Other such techniques involve the addition of noise, rotation, and scaling of the values in sequences [4]. A more advanced method, DTW Barycentric Averaging (DBA) [9], generates time-series as weighted averages of multiply aligned time-series. In that method, a time-series is selected and used for aligning the remaining samples. Then, weights that govern the alignment are iteratively updated to provide a sequence that averages the input time-series. The suitability of DBA for the training of deep learning models was discussed by Fawaz et al. [8].

The usage of data augmentation methods may depend on their application since in some domains a time-series should not be rotated and window slicing can remove vital information preventing its classification. Consequently, data warping should not be used in applications in which the time scale has significant physical meaning. Therefore, in the literature, some approaches are focused on time-series from one domain. For example, generative adversarial networks (GANs) that are composed of competing neural networks for the generation and discrimination of synthetic biosignals for the augmentation of electrocardiogram and electroencephalogram datasets were proposed by Haradal et al. [10]. A similar approach using Conditional Generative Adversarial Networks (CGAN) and irregular sampling was introduced by Ramponi et al. [13]. Biosignal recordings (electrocardiogram (ECG)) were also augmented by Cao et al. [14] using samples that belong to different categories. In that work, the deep learning methods were trained on augmented data. In other work, Delaney et al. [15] created ECG data with a range of GAN architectures. Since neural networks often require large quantities of training examples, some approaches are designed for their training. For example, Yeomans et al. [16] used smoothed curves and randomly concatenated sections from the deviation from the mean curves, Um et al. [7] perturb a temporal location of within-window event in time-series of wearable sensor data for Parkinson’s disease monitoring, or Le Guennec et al. [4] introduced noise and magnitude changes, warped or cropped time slices. Krall et al. [17], in turn, augmented electroencephalographic data using temporal and spatial or rotational distortions. The importance of time-series augmentation for healthcare applications was considered by Dahmen and Cook [18]. In that work, nested sequences obtained with hidden Markov models and regression models were used. Ruiz et al. [19] proposed a compositional synthetic signature generation from shape primitives for off-line handwritten signature verification.

In contrary to the referred methods for time-series augmentation, the method introduced in this paper, **S**ubo**P**tim**A**l **W**arped time-series ge**NE**rato**R** (SPAWNER), creates new time-series in the warped space between suboptimally aligned and merged time-series, and, as it will be seen in next sections, creates more diverse time-series and extends class boundaries.

## 3. Proposed Method

In the considered problem, a time-series $X=[x^{1},x^{2},\cdots ,x^{L}]$ is an ordered collection of *M*-dimensional values of the length *L*. Hence, each $x^{l}\in R^{M}$, l=1,2,...,L, and $X\in R^{L\times M}$.

Considering that there are *N* time-series in a dataset, and each can be of a different length $L_{n},n=1,2,...,N$, $X_{n}\in R^{L_{n}\times M}$. Then, a dataset $U=\{\left(X_{1},C_{1}\right),\left(X_{2},C_{2}\right),\cdots ,\left(X_{N},C_{N}\right)\}$ is a collection of time-series and their labels, C∈{1,K}, where *K* is the number of classes. Consequently, a classifier is trained on *U* and assigns a label *C* to a previously unseen time-series $Y\in R^{L\times M}$.

In the proposed time-series augmentation method, SPAWNER, two input sequences, $X_{1}$ and $X_{2}$, are aligned based on DTW algorithm [11]. The DTW is used as it aligns the sensor data such as voice, action, or other measurements [1,20]. However, for image or text data, other alignment methods can be more suitable [21,22]. In the introduced method, given $X_{1}=\left[x_{1}^{1},x_{1}^{2},\cdots ,x_{1}^{i},\cdots ,x_{1}^{L_{1}}\right]$ and $X_{2}=\left[x_{2}^{1},x_{2}^{2},\cdots ,x_{2}^{j},,\cdots ,x_{2}^{L_{2}}\right]$, DTW determines the optimal sequence $W=[w_{1},w_{2},\cdots ,w_{P}]$, called *warping path*, in which *P* is the length of the path, *p*-th element $w_{p}=\left(i,j\right)$, and $max\left(L_{1},L_{2}\right)\leq P<L_{1}+L_{2}$. Therefore, a $L_{1}\times L_{2}$ matrix *D* is computed. For all (i,j), it contains costs, or distances, between time-series $\left[x_{1}^{1},\cdots ,x_{1}^{i}\right]$ and $\left[x_{2}^{1},\cdots ,x_{1}^{j}\right]$. To determine the optimal alignment between $X_{1}$ and $X_{2}$, the path $W^{*}$ that minimizes the total cumulative distance is found by calculating $D\left(i,j\right)={\left(x_{1}^{i}-x_{2}^{j}\right)}^{2}+min\left(D\left(i-1,j\right),D\left(i,j-1\right),D\left(i-1,j-1\right)\right)$. Each warping path must satisfy the following three conditions:

(1) The boundary condition forces the path to start at the beginning of each time-series, $w_{1}=\left(1,1\right)$, and finish at their ends, $w_{P}=\left(L_{1},L_{2}\right)$.

(2) The monotonicity condition forces the path to contain monotonically increasing time-series indices ($i_{1}\leq i_{2}\leq \cdots \leq L_{1},j_{1}\leq j_{2}\leq \cdots \;\leq L_{2}$).

(3) The continuity condition, which also implies the monotonicity, can be written as $w_{p+1}-w_{p}\in \{\left(1,0,\left(0,1\right),\left(1,1\right)\right\}\forall_{p\in \{1,2,\cdots ,P-1\}}$. However, to reduce computational costs and align parts of time-series that are close to each other in time, an additional constraint is often used [23].

Specifically, the warping window ξ limits the elements of $X_{1}$ and $X_{2}$ that can be aligned, i.e., $\forall_{\left(i,j\right)\in w_{p}}||i-j||\leq \xi$. Typically, ξ is 10 percent of $max(L_{1},L_{2})$ [11]. Since DTW is used for the distance computation between time-series, the value $D(L_{1},L_{2})$ is returned.

In this work, to create new time-series given two input examples $X_{1}$ and $X_{2}$, an additional constraint on the warping path is introduced forcing it to contain the element $w_{p}=\left(R_{1},R_{2}\right)$, where $R_{1}=\left\lceil rL_{1}\right\rceil ,R_{2}=\left\lceil rL_{2}\right\rceil$, *r* is a single uniformly distributed random number in the interval (0,1) and ⌈·⌉ is the ceiling operator.

However, to prevent the calculation of $L_{1}\times L_{2}$ matrix *D* and reduce the computational cost, two matrices $R_{1}\times R_{2}$ $D_{1}$ and $|\left(L_{1}-R_{1}\right)\left|\times \right|\left(L_{2}-R_{2}\right)|$$D_{2}$ are used. Then, $\left[x_{1}^{1},x_{1}^{2},\cdots ,x_{1}^{R_{1}}\right]$ is aligned with $\left[x_{2}^{1},x_{2}^{2},\cdots ,x_{2}^{R_{2}}\right]$ and $\left[x_{1}^{R_{1}+1},x_{1}^{R_{1}+2},\cdots ,x_{1}^{L_{1}}\right]$ is aligned with $\left[x_{2}^{R_{2}+1},x_{2}^{R_{2}+2},\cdots ,x_{2}^{L_{2}}\right]$. Consequently, warping paths $W_{1}^{*}$ and $W_{2}^{*}$ are obtained. The paths are optimal, taking into account the introduced constraint and matrices $D_{1}$ and $D_{2}$ but after their concatenation the obtained path is suboptimal considering the path that can be obtained for *D*.

Furthermore, as DTW aligns both sequences using a relatively great ξ and, in the proposed method, $\xi_{1}$ and $\xi_{2}$ used to determine $W_{1}^{*}$ and $W_{2}^{*}$ depend on the value of $\left\lceil 0.1\cdot max\left(R_{1},R_{2}\right)\right\rceil$ and $\left\lceil 0.1\cdot max(\right|L_{1}-R_{1}\left|,\right|L_{2}-R_{2}\left|)\right\rceil$, respectively. These values reduce the flexibility of the path from the perspective of the matrix *D* as well as the concatenated paths $W_{1}^{*}$ and $W_{2}^{*}$.

Once the concatenation of the paths is obtained ($W_{1,2}^{*}$), the method aligns $X_{1}$ to $X_{2}$ generating sequences $X_{1}^{⋆}$ and $X_{2}^{⋆}$ of the length of $W_{1,2}^{*}$. In SPAWNER, to produce a new time-series $X^{⋆}$, $X_{1}^{⋆}$ and $X_{2}^{⋆}$ are merged. Here, their average was primarily considered. However, as observed for many multivariate time-series examples, in some dimensions, values may not change much in time and an introduction of a slight changes around the average facilitates the application of classifiers that depend on signal variances [24]. Therefore, a value $x^{⋆}\in X^{⋆}$, is a random number chosen from a normal distribution with a small σ to draw values close to the average, $x^{⋆}∼N\left(\mu ,\;\sigma^{2}\right)$, $\mu =0.5\left(x_{1}^{⋆}+x_{2}^{⋆}\right),\sigma =0.05\left|x_{1}^{⋆}-x_{2}^{⋆}\right|$.

**Figure 1:**
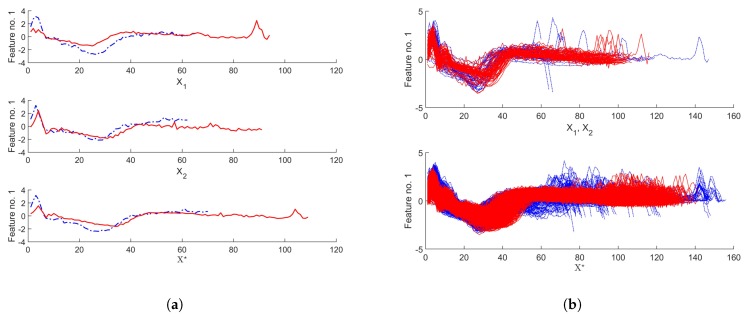
Augmented time-series from *ECG* dataset [25] using the proposed method. The first two rows show input time-series of two classes, the third row presents new examples (**a**). The plot for the entire ECG dataset highlights the variability of augmented time-series (**b**). Classes are indicated by colors.

To present the output of the method, two time-series from *ECG* dataset [25] of each class for an exemplary feature dimension are shown in Figure 1a along with the time-series obtained with SPAWNER. The classes are indicated by colors. The figure also contains augmented data for all input sequences (Figure 1b). The method creates one time-series for each input pair that belong to the same class.

The method was implemented in Matlab R2018b using Signal Processing Toolbox on the i7-6700K 4 GHz CPU with 64 GB RAM. Its code and exemplary application are available at http://marosz.kia.prz.edu.pl/SPAWNER.html.

## 4. Results and Discussion

### 4.1. Datasets

In experiments, ten multivariate time-series benchmark datasets are employed. They represent a variety of collected sensor data, including challenging time-series from activity recognition datasets with common nonlinear distortions in time scale. The computation time with the augmented dataset with some of the applied classifiers on the used processing unit was a factor considered when selecting the benchmarks for the experiments. The benchmarks are described in Table 1, most of them contain time-series of different lengths (cf. Figure 1), with the number of classes in benchmarks varied from 2 to 95, and the number of attributes from 2 to 75. Furthermore, some of them contain a relatively small number of examples (e.g., *Kick* vs. *Punch*, *Occupancy*, or *AREM*).

### 4.2. Time-Series Augmentation Methods

The proposed method is experimentally compared with three representative approaches: DBA [9], *window slicing* (WS) and *window warping* (WW) [4]. The Matlab sourcecode of DBA is publicly available while the remaining methods are implemented by the authors of this paper based on findings published by Le Guennec et al. [4]. Among the compared approaches, DBA averages time-series and takes into account nonlinear transformations in time scale, reflected by different lengths of sequences in a dataset. In experiments, DBA provides average time-series for a randomly selected subset of the training data samples until the targeted size of the dataset is achieved. In the WW, randomly selected part of a time-series of 10% of its length is warped [4], while in WS, a slice is removed from the sequence. Both data augmentation techniques are simple but considering the simplicity of image operators typically used for image augmentation in computer vision applications, their performance should be examined. For a given dataset, its time-series are selected and processed until the required size of the output dataset is met. The input examples are added to the generated examples. Since SPAWNER generates new data from a pair of input time-series, the number of generated examples by other methods is based on the size of the produced output dataset to ensure a fair comparison of approaches. The number of data samples per dataset used in the evaluation is also given in Table 1.

### 4.3. Time-Series Classifiers

The compared data augmentation methods generate training data for three time-series classifiers: nearest neighbor (NN) with DTW distance (NN-DTW), LogDet divergence-based metric learning with triplet constraints (LDMLT) [28], and the nearest neighbor with recently introduced time-series cluster kernel (NN-TCK) [24]. These three classifiers are employed in tests due to their popularity and the state-of-the-art performance. Also, their Matlab sourcecodes are publicly available and their efficient classification of large time-series datasets does not require specific hardware setting [29]. In the implementation of the NN-DTW classifier, the size of the warping window is equal to the 10% of the length of the longer sequence from the pair of sequences for which the DTW distance is calculated. Then, the distance is normalized by the sum of time-series’ distances to reduce the impact of short time-series on the calculated distance. Since TCK cannot provide the kernel for time-series of different lengths, in experiments with its use, multidimensional time-series are transformed into time-series of the same length according to the approach of Wang et al. [30], also employed by Mikalsen et al. in tests with TCK [24]. The length of the resulted time-series is equal to $\left\lceil T_{max}/\left\lceil T_{max}/25\right\rceil \right\rceil$, where $T_{max}$ is the length of the longest time-series in a dataset. In experiments, the time-series are standardized to zero mean and unit standard deviation [24].

It is worth noticing that the classification performance of LDMLT and NN-TCK with augmented time-series is reported for the first time in this paper and most studies on such augmentation consider only one classifier.

### 4.4. Time-Series Classification with Augmented Datasets

In the evaluation, augmentation methods are compared using average classification accuracy and the average and geometric average ranks for methods and classifiers.

The accuracy is calculated as the number of correct predictions of the classifier divided by the total number of testing samples in the dataset. The division of a dataset into training-testing subsets is shown in Table 1. The average accuracy is calculated for 50 runs of the data augmentation methods in experiments with NN-DTW and 10 runs with the remaining classifiers due to their high computational demand (NN-TCK, LDMLT). To compare augmentation methods ranks from 1 to 5 are used, where a lower rank is assigned to the method with greater accuracy. The results are presented in Table 2.

The experimental comparison reveals that all three classifiers display a variety of performances on the benchmark datasets in a case in which data augmentation methods are not used. Specifically, each of them is better than the remaining two for some benchmarks. Here, NN-DTW outperforms NN-TCK and LDMLT on five datasets (*Gesture phase*, *EEG*, *Movement AAL*, *Ozone*, and *LIBRAS*), NN-TCK exhibits the best performance on three datasets (*ECG*, *AREM*, and *Occupancy*), and LDMLT on two (*AUSLAN* and *Kick vs. Punch*). The compared augmentation methods can improve the classification accuracy four times for NN-DTW and two times for NN-TCK. Apart from the improvement of the best-obtained results for the classifiers, the augmentation techniques also allowed them to improve their remaining results in many cases. The introduced SPAWNER outperforms other such techniques in terms of the number of datasets for which the accuracy is improved. Also, it is better ranked.

Interestingly, the compared methods seem less influential on the results of the NN-TCK classifier, due to the way it processes the time-series (i.e., the interpolation to shorter sequences). Taking into account the results for the datasets, it can be seen that the performance of a classier for *Kick vs. Punch* dataset does not change with synthetic examples and for *EEG* dataset it dropped for NN-DTW and LDMLT. For *EEG* benchmark, it is caused by close proximities of samples from different classes and the introduction of synthetic examples in areas that belong to a different class is such scenarios. For *Kick vs. Punch*, in turn, most of the training examples are separated from each other and new examples do not bring information that could be used by a classifier to correctly assign testing examples. However, the augmentation methods improved the baseline results eight times for NN-DTW and LDMLT and six times for NN-TCK. This justifies the development of the synthetic time-series generation approaches. The visualization presented in Section 4.5 highlights these problems and further shows differences between augmentation techniques.

### 4.5. Visualization

The accuracy of the augmentation methods reflects the way the synthetic samples appear in the space among input samples. This can be shown using Multi-Dimensional Scaling (MDS) [31] that reduces the data dimensionality and places samples in new space, using pairwise dissimilarities between them. However, the pairwise DTW distances cannot be used in this case, since DTW is not a metric. Therefore, in this work, Kruskal’s nonmetric MDS was employed that approximates the ranks of the dissimilarities. Specifically, the obtained visualization contains points and distances between them, approximating a monotonic transformation of the samples’ dissimilarities [31]. Hence, it can be used to interpret the relationship between samples of time-series in a dataset.

**Figure 2:**
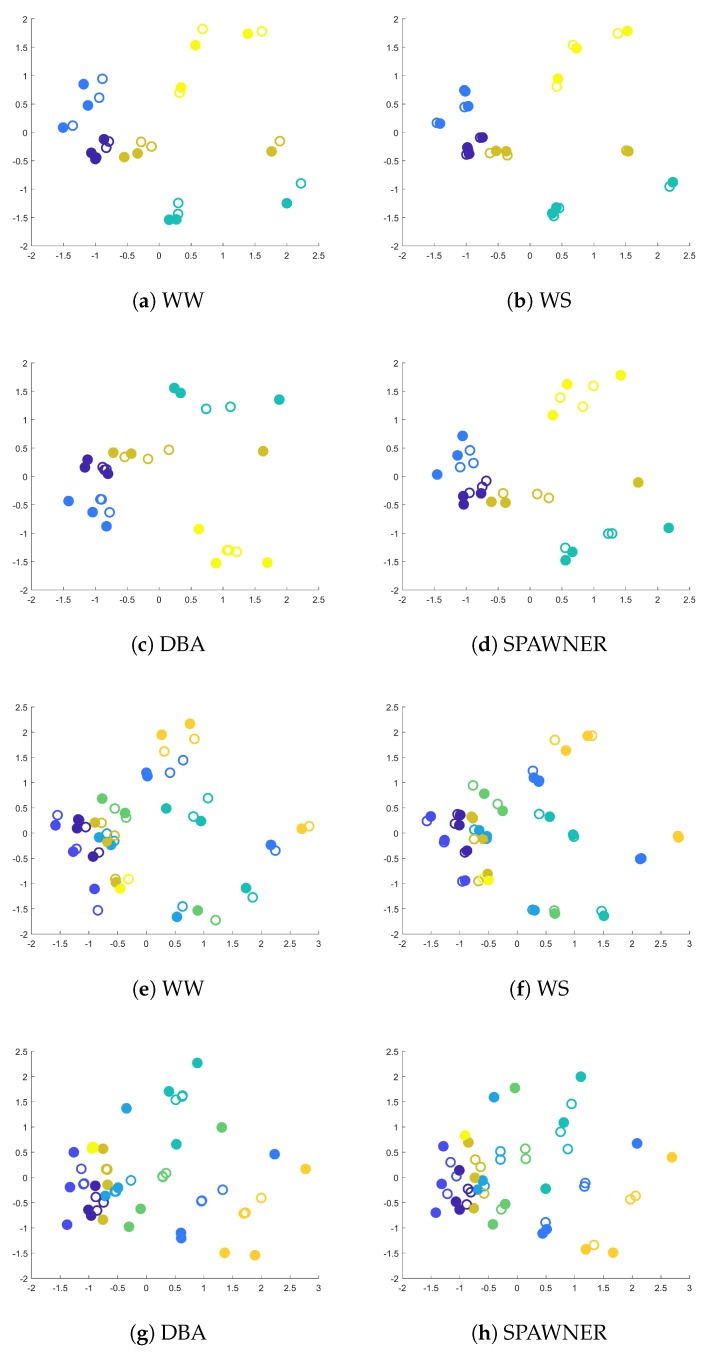
Scatter plots with two-dimensional MDS embedding of DTW dissimilarities between the first 15 (the first two rows) or 25 (the last two rows) training sequences from *AUSLAN* dataset, including augmented time-series generated by four methods. In the plots, the classes are denoted by colors, the filled circles denote input time-series, and the circles with white interior indicate synthetic samples.

The scatter plots containing MDS representations of the first 15 and 25 sequences and the resulted augmented samples from *AUSLAN* dataset are shown in Figure 2. The number of considered sequences is low to facilitate the visualization. The case in which more samples are used (i.e., Figure 2e–h) is presented to highlight the difficulty of the classification task in which more similar samples from different classes are taken into consideration. Larger or smaller dissimilarities, reflected by DTW distances, correspond to larger or smaller Euclidean distances between points in plots. As shown, WW and WS tend to create new samples close to the original time-series, as they share most of their parts. This can be beneficial in tight spaces between classes (Figure 2e,f) where samples from different classes are close to each other and may confuse a classifier. However, for some datasets, they are unable to create samples that would use space between original sequences. Here, DBA and SPAWNER, put new samples in space between input sequences, often creating new boundaries that can be closer in the nearest neighbor sense than it can be observed for the original data. Also, the diversity of the obtained time-series for these methods is larger in comparison to WW and WS methods. In contrary to DBA, the introduced SPAWNER does not concentrate samples in the center of the cluster for a class (cmp. Figure 2c,d). In such cases, the generation of new samples does not bring an additional advantage, since input samples cover the area. Some input sequences are introduced that are closer to other classes than to the rest of the samples from their class. Here, SPAWNER create samples that connect the outlier sequence to its class, DBA seems to use the sequence to create a new one that slightly shifts the class boundary in the direction towards the outlier, and warping with slicing techniques create sequences in its neighborhood. This can be seen for horizontally placed examples of the “green” class shown in Figure 2a–d. Interestingly, with additional samples from the dataset, the plots begin to show the difficulty of the classification task caused by the similarities between classes (Figure 2e–h) and new class boundaries created by SPAWNER and DBA.

### 4.6. Time-Series Classification with Augmentation Based on Few Examples

The results reported in previous experiments reveal that data samples in a benchmark are often closer to examples of different classes, contributing to worse performance of a classifier. Consequently, the obtained synthetic examples when establishing new class boundaries may introduce samples in places that belong to other classes. However, for most datasets, new examples improved classification accuracy.

**Figure 3:**
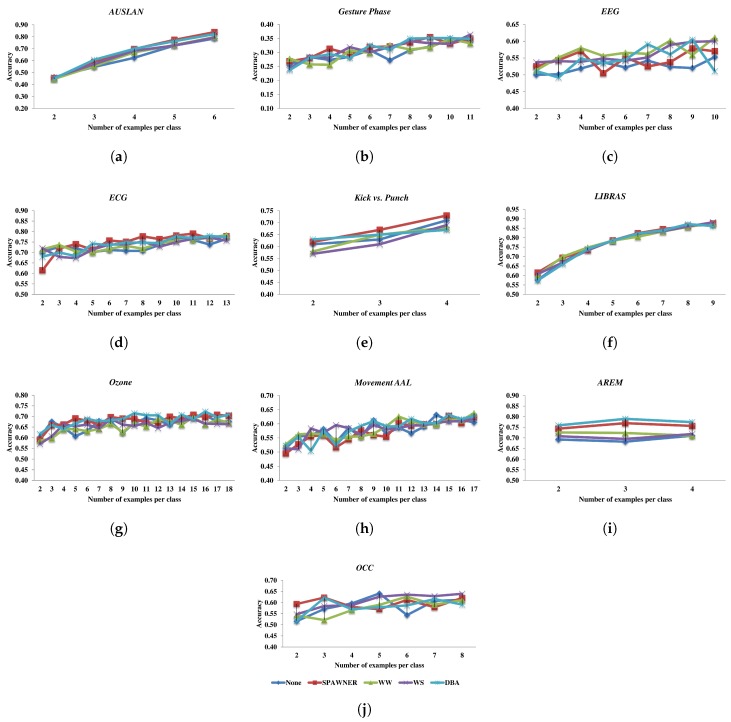
Accuracy of NN-DTW classifier with augmented small number of examples per class.

Since some datasets may contain only a few examples, the usability of the time-series augmentation method introduced in this paper is examined on benchmarks with a reduced number of samples. For each dataset, randomly selected samples are used for the augmentation. In the experiments, their number varied from two to the size that allows a time-series generation method to produce the dataset that doubles the size of the original dataset. The input examples are added to the generated examples. To compare data augmentation algorithms, the average classification accuracy of the NN-DTW method is used on ten benchmark datasets, taking into account ten runs for each number of examples per class. This classifier is used in tests as the number of performed experiments and the size of the training data make the application of the remaining classifiers unfeasible. The results are presented in Figure 3.

As shown, the addition of more examples per class improves the classification performance but it locally depends on the randomly selected examples. Interestingly, the accuracy obtained with a small number of input samples augmented by SPAWNER and DBA for *AREM* dataset is greater than the value obtained for the entire dataset due to close boundaries between classes present in the entire (i.e., original) dataset or a large number of outliers. Also, almost in all experiments with the augmentation, the accuracy is improved. This means that for small datasets the generation of new samples is justified and should be employed. Since the comparison based on data presented in Figure 3 can be difficult due to the amount of conducted experiments, the approaches are compared using average accuracy per dataset. The results are shown in Table 3.

From the results of experiments with a small number of samples per benchmark, it can be seen that the introduced SPAWNER clearly outperforms other techniques, as it allowed the classifier to obtain the best result for a dataset five times, and is ranked best across datasets. The values for the case in which augmentation is not used (cf. the second column in the table) also confirm that the augmentation is beneficial if only a few examples are available.

### 4.7. Complexity Analysis

To compare the complexity of the time-series augmentation methods, their time- and memory- consumption on a representative dataset is evaluated. In experiments, LIBRAS dataset is used since it contains a reasonably large number of classes. The average processing time and memory requirements are determined on a basis of 10 runs of the methods. The results shown in Table 4 indicate that WW and WS are much less complex than DBA and SPAWNER. However, since SPAWNER uses DTW to align pairs of time-series and DBA aligns all selected time-series similarly to DTW, SPAWNER requires fewer resources and generates augmented sequences faster than DBA.

### 4.8. Limitations of the Method

Since the proposed method constrains the warping path while aligning two sequences to produce a new time-series, the case in which the warping path is not constrained is investigated. The average accuracy of the NN-DTW classifier calculated for 50 runs of SPAWNER for both cases is reported in Table 5. This classifier is used in tests since the time-series generated by the method influence its results the most. In the table, maximal values obtained for the constrained method (i.e., suboptimal) are also reported.

As reported, the classifier with the proposed method of constraining the path outperforms the option in which such a constraint is not used for six datasets. For one dataset, the results were not changed, while in three cases the constraint resulted in a drop in the accuracy. Taking into account the best results obtained by the introduced approach, its application is justified in all cases. This means that the observed results for the aforementioned three datasets can be obtained by the classifier with the applied method using the constrained warping paths. Consequently, it can be assumed that there may be a way of aligning the time-series to provide satisfactory results for each benchmark dataset.

**Figure 4:**
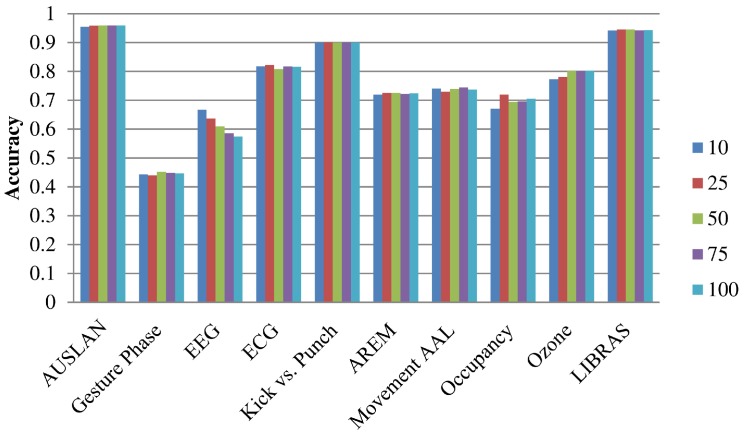
Accuracy of NN-DTW classifier with augmented time-series generated by SPAWNER considering a different number of neighboring sequences in the selection of input sequence pairs. The number of selected time-series is given in percent.

Since the augmentation proposed in this paper does not consider a rejection step of input outliers that may decrease the quality of the augmented time-series, a time-series selection is introduced that allows a given input time-series creating output sequence only with some of its neighbors. The results in terms of the average accuracy for 50 runs of the NN-DTW classifier for all datasets ranging the number of neighbors from 10 to 100% of input sequences are shown in Figure 4. As shown for all datasets, excluding the EEG which does not respond well to the augmented data, the access to more neighbors while creating new samples using input pairs is beneficial to the performance of the classifier. Consequently, it seems that the datasets do not contain outliers decreasing its performance and the more distant time-series should be involved in the time-series augmentation.

## 5. Conclusions

In this work, the problem of generating artificial examples to augment time-series datasets is considered. A new method that aligns the input time-series and constrains the warping path is proposed. The proposed modification of the warping path while aligning the sequences allows producing new examples that resemble the original data. It is shown that the produced synthetic examples are better utilized by three time-series classifiers than those of other related methods, improving their classification accuracy on the demanding ten benchmark datasets. Furthermore, the proposed method creates more diverse examples and moves class boundaries.

In future work, deep learning architectures for time-series classification [32], data augmentation near class boundaries, or augmentation addressing a specific domain (e.g., captured human actions or sign language gestures [33,34]) will be considered.

To facilitate the reproducibility of the presented findings, the code of the introduced SPAWNER is available at http://marosz.kia.prz.edu.pl/SPAWNER.html.

## Figures and Tables

**Table 1:** Time series benchmark datasets used in experiments.

Name	Research	Classes	Attributes	Max	Train-Test	Training	Augmented
	Area			Length	Split	Samples	Samples
AUSLAN [26]	Sign Language Recognition	95	22	96	44-56	1140	6270
Gesture Phase [26]	Gesture Recognition	5	18	214	50-50	198	4142
EEG [26]	EEG Classification	2	13	117	50-50	64	996
ECG [25]	ECG Classification	2	2	147	50-50	100	2706
Kick vs. Punch [27]	Action Recognition	2	62	761	62-38	16	57
AREM [26]	Activity Recognition	7	7	480	50-50	43	132
Movement AAL [26]	Movement Classification	2	4	119	50-50	157	6084
Occupancy [26]	Occupancy Classification	2	5	3758	35-65	41	400
Ozone [26]	Weather Classification	2	72	291	50-50	172	7635
LIBRAS [26]	Sign Language Recognition	15	2	45	38-62	360	4278

**Table 2:** Experimental comparison of augmentation methods using three time-series classifiers in terms of the classification accuracy. The greatest value used in the rank is written in bold and rounded to two significant figures.

Dataset/Aug. Method	None	SPAWNER	WW	WS	DBA
NN-DTW					
AUSLAN	0.92	**0.96**	0.92	0.92	0.95
Gesture Phase	0.42	**0.45**	0.43	0.43	0.43
EEG	**0.75**	0.57	0.67	0.68	0.55
ECG	0.82	0.82	0.81	0.80	**0.84**
Kick vs. Punch	**0.90**	**0.90**	**0.90**	**0.90**	**0.90**
AREM	0.72	0.72	0.72	0.72	**0.79**
Movement AAL	0.73	0.74	0.74	**0.75**	0.73
Occupancy	0.64	**0.71**	0.57	0.70	0.69
Ozone	0.79	**0.80**	0.78	0.80	0.80
LIBRAS	0.95	0.94	0.95	**0.96**	0.95
NN-TCK					
AUSLAN	**0.71**	0.57	0.09	0.31	0.01
Gesture Phase	0.17	**0.30**	0.17	0.17	0.17
EEG	0.48	0.50	**0.57**	0.54	0.48
ECG	**0.88**	0.84	0.77	0.80	0.80
Kick vs. Punch	**0.52**	0.40	0.40	0.40	0.40
AREM	0.72	**0.91**	0.81	0.78	0.18
Movement AAL	0.65	0.66	**0.68**	0.67	0.64
Occupancy	0.68	0.66	0.67	0.64	**0.70**
Ozone	**0.55**	0.39	0.39	0.39	0.39
LIBRAS	0.94	0.93	**0.94**	0.94	0.94
LDMLT					
AUSLAN	**0.98**	0.97	0.96	0.96	0.97
Gesture Phase	0.31	**0.41**	0.29	0.29	0.41
EEG	0.70	0.61	0.69	**0.70**	0.57
ECG	0.82	0.80	**0.82**	0.81	0.82
Kick vs. Punch	**1.00**	**1.00**	**1.00**	**1.00**	**1.00**
AREM	0.69	0.72	0.67	0.68	**0.77**
Movement AAL	0.66	0.69	0.66	**0.70**	0.67
Occupancy	0.57	**0.75**	0.70	0.65	0.65
Ozone	0.67	0.73	0.65	0.00	**0.74**
LIBRAS	0.94	**0.94**	0.94	0.94	**0.94**
Overall results					
Count best	8	**11**	6	6	8
Average rank	2.98	**2.60**	3.30	3.02	3.10
Geometric average rank	2.64	**2.23**	2.94	2.73	2.73

**Table 3:** Experimental comparison of augmentation methods using NN-DTW classifier and few number of examples per class. Average accuracy for a dataset is reported (see Figure 3). The greatest value used in the rank is written in bold and rounded to two significant figures.

Dataset/Aug. Method	None	SPAWNER	WW	WS	DBA
AUSLAN	0.63	**0.67**	0.64	0.65	0.67
Gesture Phase	0.30	**0.32**	0.30	0.31	0.31
EEG	0.52	0.55	**0.57**	0.56	0.54
ECG	0.73	**0.75**	0.74	0.73	0.74
KickvsPunch	0.65	**0.67**	0.64	0.62	0.65
AREM	0.69	0.76	0.72	0.71	**0.77**
Movement AAL	0.58	0.57	0.58	0.58	**0.58**
Occupancy	0.58	0.60	0.58	**0.61**	0.58
Ozone	0.66	0.68	0.65	0.66	**0.68**
LIBRAS	0.77	**0.78**	0.78	0.77	0.77
Overall results					
Count best	0	**5**	1	1	3
Average rank	4.15	**1.9**	3.3	3.2	2.45
Geometric average rank	4.03	**1.61**	2.99	2.96	2.09

**Table 4:** Time- and memory-consumption of compared time-series augmentation methods.

Method	Computation Time (s)	Storage (MB)
SPAWNER	3.23	1.23
WW	0.01	0.06
WS	0.01	0.07
DBA	49.6	7.18

**Table 5:** Influence of the introduced constraint on the average performance of NN-DTW classifier with augmented data generated by SPAWNER. The best result for each benchmark dataset is written in bold and rounded to two significant figures.

Dataset/Method	Constrained (Suboptimal)		Unconstrained
	Mean	Maximum	(Optimal)
Auslan	0.96	**0.96**	0.96
Gesture Phase	0.45	**0.46**	**0.46**
EEG	0.57	**0.61**	0.56
ECG	0.82	**0.84**	0.81
Kick vs. Punch	**0.90**	**0.90**	**0.90**
AREM	0.72	**0.74**	**0.74**
Movement AAL	0.74	**0.77**	0.70
Occupancy	0.71	**0.74**	0.71
Ozone	0.80	**0.84**	0.72
LIBRAS	0.94	**0.95**	0.94
